# BactImAS: a platform for processing and analysis of bacterial time-lapse microscopy movies

**DOI:** 10.1186/1471-2105-15-251

**Published:** 2014-07-25

**Authors:** Igor Mekterović, Darko Mekterović, željka Maglica

**Affiliations:** Department of Applied Computing, Faculty of Electrical Engineering and Computing, University of Zagreb, Unska 3, 10000 Zagreb, Croatia; Division of Experimental Physics, Ruđer Bošković Institute, Bijenička cesta 54, 10000 Zagreb, Croatia; School of Life Sciences, Swiss Federal Institute of Technology in Lausanne (EPFL), 1015 Lausanne, Switzerland

**Keywords:** Time-lapse microscopy, Mycobacteria, Image analysis, ImageJ, Icy, Data visualization, Database

## Abstract

**Background:**

The software available to date for analyzing image sequences from time-lapse microscopy works only for certain bacteria and under limited conditions. These programs, mostly MATLAB-based, fail for microbes with irregular shape, indistinct cell division sites, or that grow in closely packed microcolonies. Unfortunately, many organisms of interest have these characteristics, and analyzing their image sequences has been limited to time consuming manual processing.

**Results:**

Here we describe BactImAS – a modular, multi-platform, open-source, Java-based software delivered both as a standalone program and as a plugin for Icy. The software is designed for extracting and visualizing quantitative data from bacterial time-lapse movies. BactImAS uses a semi-automated approach where the user defines initial cells, identifies cell division events, and, if necessary, manually corrects cell segmentation with the help of user-friendly GUI and incorporated ImageJ application. The program segments and tracks cells using a newly-developed algorithm designed for movies with difficult-to-segment cells that exhibit small frame-to-frame differences. Measurements are extracted from images in a configurable, automated fashion and an SQLite database is used to store, retrieve, and exchange all acquired data. Finally, the BactImAS can generate configurable lineage tree visualizations and export data as CSV files. We tested BactImAS on time-lapse movies of *Mycobacterium smegmatis* and achieved at least 10-fold reduction of processing time compared to manual analysis. We illustrate the power of the visualization tool by showing heterogeneity of both *icl* expression and cell growth atop of a lineage tree.

**Conclusions:**

The presented software simplifies quantitative analysis of time-lapse movies overall and is currently the only available software for the analysis of mycobacteria-like cells. It will be of interest to the community of both end-users and developers of time-lapse microscopy software.

**Electronic supplementary material:**

The online version of this article (doi:10.1186/1471-2105-15-251) contains supplementary material, which is available to authorized users.

## Background

Multiple cellular properties can be monitored simultaneously over time at the single-cell level using fluorescence time-lapse microscopy [1, 2]. This method is based on the repeated imaging of cell(s) at regular time intervals, resulting in an image sequence that can be viewed as a time-lapse movie. By combining it with a computer controlled microscope stage and microfluidic devices, many cells can be observed in controlled, varying environments for days [3, 4]. This powerful method is currently the only available approach to obtain real-time information on the dynamics of intracellular processes, to determine individual cell lineages, and to monitor cell-to-cell variation over time.

Quantitative biological information needs to be extracted through subsequent processing of time-lapse movies, a step that significantly limits the potential of this method. This entails delineating individual cells in each movie frame (segmentation), following identified cells through the movie (tracking), and identifying cell division events (lineage). Done manually, this process is extremely time-consuming and error-prone, whereas universal and fully automated software does not exist. Nevertheless, many automated analysis programs have been developed for specific experimental setups, mostly for analysis of eukaryotic cells (for review see [5]). For prokaryotic cells, several software solutions exist [6–11], among which only Schnitzcells [6] and MicrobeTracker [7] are widely used.

All these programs were developed and tested for the model organisms *Escherichia coli*, *Bacillus subtilis* or *Caulobacter crescentus* under a limited set of experimental conditions, but were also used with other bacteria having similar cell properties [12–14]. All these bacterial species share low variation in shape and size between individual cells and exhibit clear contours, even when allowed to grow into a large microcolony. These characteristics enable existing algorithms to recognize every cell in each frame. However, some bacterial types cannot be easily segmented. Specifically, none of the known programs could be used to automate analysis of mycobacterial time-lapse movies. This is attributed to the tendency of mycobacterial cells to form closely-packed microcolonies, lacking visible separation between neighbors. Moreover, individual cells possess highly irregular morphology and do not form readily detectable division sites. These factors, combined with technical issues and experimental conditions that sometimes result in poor image quality, preclude automated analysis.

Development of image analysis software that can facilitate processing of mycobacterial cells would be of great value to the field. The Mycobacterium genus notably includes *Mycobacterium tuberculosis*, the causative agent of tuberculosis. This disease caused over 1.4 million deaths in 2011 and the emergence of drug-resistant strains poses a great public health threat and global economic burden [15]. Several recent publications have demonstrated that it is possible to monitor real-time growth of *M. tuberculosis* and its non-pathogenic relative *M. smegmatis* by combining fluorescence time-lapse microscopy and microfluidics. These studies have resulted in new biological insights on cell cycle dynamics, antibiotic persistence, and drug-susceptibility [4, 16–19].

In all these studies, mycobacterial time-lapse movies were analyzed manually by using multipurpose image analysis platforms such as ImageJ [20]. However, none of these analyses were real-time gene expression studies and hence did not require the tracking of each individual cell in every frame of the time-lapse movie. Such gene-expression analysis would generate considerably larger and more complex datasets and it would become challenging to efficiently store, exchange, statistically analyze, and visualize them. Therefore, beyond the problem of cell segmentation and tracking algorithms, a major obstacle to a much wider application of bacterial time-lapse microscopy is processing and managing the generated data. This is, in fact, a common problem to all bioimaging methods, see [21].

In this study we present BactImAS (BACTerial IMage Analysis Software) – a Java-based program designed for semi-automated cell segmentation and tracking, as well as storage, analysis, and visualization of acquired time-lapse data. It includes a newly developed algorithm that allows segmentation of mycobacterial cells and manages extracted data using a database. We tested BactImAS on time-lapse movies from an *M. smegmatis* ICL reporter strain and analyzed expression dynamics of the *icl* gene using a novel visualization tool and a built-in SQL editor.

## Implementation

**Figure 1:**
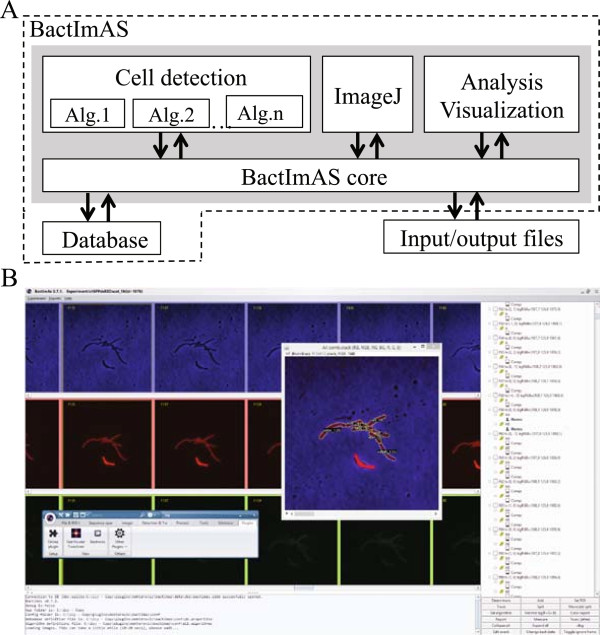
**Program architecture and GUI. (A)** Schematic representation of BactImAS modular architecture. The gray box represents the main, Java-based part of BactImAS. The database (SQLite) is a separate part of the platform. All communication between different modules, indicated with arrows, is done through the BactImAS core. **(B)** BactImAS GUI: Panel on the right displays a frame →*bacteria*→ROI hierarchy of the current movie and is synchronized with three horizontal scroll panes on the left, showing recorded images from each channel. Selected frame with annotated segmented cells is shown using an ImageJ stack dialog (containing all image channel combinations). The user can manipulate selections using the incorporated ImageJ application. Status pane on the bottom displays various messages from the program e.g. progress updates, measurements, etc.

BactImAS is a Java-based program that incorporates the latest ImageJ version (currently 1.49a) [20] and a relational database (SQLite3) (Figure 1A). The interaction between the program and the user is facilitated through a graphical user interface loosely inspired by movie editing tools (Figure 1B). We incorporated ImageJ, a tool well known to the biological community, in our GUI for various image processing tasks. We also used ImageJ data structures and functionalities in the implementation of our algorithm. The program is distributed, with identical features, in two ways: as an Icy [22] plugin and as standalone software. If used as a plugin, BactImAs is installed (and kept up to date) through Icy. Installation of the standalone version consists of downloading and extracting the application files from [23]. In both cases, the SQLite database has to be installed for the program to work. In Windows OS, SQLite installation is a simple task, whereas in Mac OS X and many distributions of Linux OS it is already pre-installed.

Analyzing time-lapse movies with BactImAS consists of preprocessing the images, followed by computer-assisted segmentation and tracking, and finally measurement of features of the segmented cells for further analysis. All regions of interest (ROIs) and extracted measurements are automatically and continuously saved in the database, thereby reducing the chance of error and data loss. This feature also allows image processing to be a collaborative effort between multiple users.

### Preparation of images

Individual images from up to three recorded channels must be stored as image sequences. Alongside the set of original images, the user has to provide the 8-bit PNG versions (selected because of their small file size) that the software uses internally for displaying and tracking tasks. Both these sets are easily prepared using, for instance, the attached ImageJ program. This does not limit the format of the original images, as measurements can be taken on any image format supported by ImageJ.

Due to the imprecision of the motorized stage, there is often a translational shift between two successive frames in an image sequence. This must be corrected before cell segmentation. To this end, BactImAS has a simple and robust built-in registration algorithm (see Additional file 1) to identify and correct this. If this algorithm fails, images can be registered using external software.

Apart from these mandatory steps, the user may opt to define parameters such as pixel-to-micron ratio, specify frames that should be ignored (e.g. out-of-focus frames), and associate experimental information with the corresponding frame (e.g. change of growth medium).

### Cell segmentation and tracking

Once the images are loaded, the user must delineate contours of the cell(s) of interest in the first frame. For easier tracking of cell poles, generations, and lineages in general, initial cells are named in an alphabetical manner and their progenies in binomial fashion (e.g. cell A divides to AA and AB, cell AB then divides into ABA and ABB etc). The user then selects an algorithm that segments and tracks each cell for the assigned number of frames on a selected channel.The user can easily inspect the segmentation results throughout the movie processing step. Upon selecting a frame of interest, the program produces a new image stack with ROI selections superimposed on the images from each channel (e.g. blue, green, and red) and of merged combinations of channels (B+G, B+R, G+R, B+G+R) (Figure 1B). In addition, BactImAS can generate an image sequence of all processed frames composed of ROI selections superimposed on phase-contrast images. If errors are observed, the segmentation can be repeated with different algorithm parameters or by using images from a different channel (if available). Alternatively, any erroneously segmented cells can be manually corrected. This not only corrects the ROI selection of the selected frame, but also facilitates the correct segmentation for the subsequent frames.

In addition to manual selection of cells, it is possible to set a specific cell to be ignored (e.g. if a tracked cell partially exits the image borders), to be defined as “dead”, or to be assigned any other property from a certain frame onwards.In many cell types, a division event is easily defined by a membrane constriction between two adjacent cells, visible on phase-contrast images. In contrast, in mycobacteria a division event often precedes any identifiable membrane constrictions. This mycobacterial feature makes it challenging for an algorithm to define the exact frame and intracellular position of the division event. BactImAS therefore relies on the user to define the division events throughout movie processing. The user is assisted in this decision-making process in two ways: image sequences of each channel are separately displayed in the GUI and the aforementioned image stack, displaying a combination of channels for a selected frame (Figure 1B). After recognizing a division event, the user has to define which specific cell divided (this automatically generates a name for each daughter cell and allows the program to keep track of cell lineages) and then to delineate the contours of the two daughter cells.

### Mycobacterial segmentation algorithm

**Figure 2:**
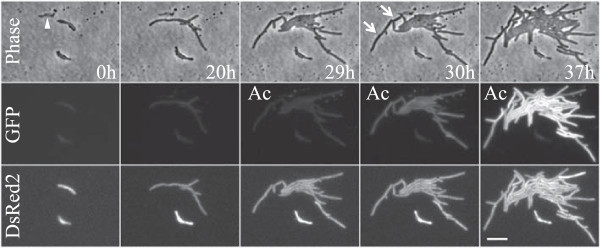
**Snapshots of mycobacterial time-lapse movie.** Images of phase-contrast, green fluorescence, and red fluorescence channels taken at indicated times from the time-lapse movie following the reporter strain of *M. smegmatis*. GFP signal, representing induction of the *icl* gene, increases upon addition of acetate (Ac), whereas the constitutively-expressed DsRed2 signal is present throughout the experiment. Main challenges of automated analysis of such a movie are noted: lack of visible edges between adjacent cells, lack of clear division site, cell shape variations (arrows), and non-cell objects present in the images (arrowhead). Scale bar, 5 *μ*m.

Previously developed algorithms cannot deal with typical *M. smegmatis* time-lapse movie due to challenges illustrated in Figure 2. To segment mycobacteria-like cells we developed an algorithm (for detailed description see Additional file 1) based on the following assumptions: cells change only slightly from frame to frame thus making tracking straightforward; cells are sometimes in close juxtaposition to neighboring cells but most juxtaposed cells have at least some segments of visible edge; cells are worm-shaped with a relatively fixed width but varying length; and almost all growth happens along the length of the cell.

**Figure 3:**
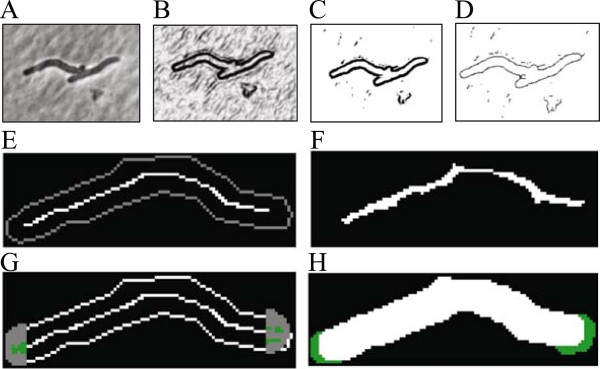
**Description of the algorithm. (A)** Original image. **(B)** Image after edge detection. **(C)** Thresholding. **(D)** Skeletonization. **(E)** Cell selection from the previous frame thinned to the skeleton pixels (white line). **(F)** Pixels of the adjusted skeleton area for the skeleton in E. **(G)** Area probed for elongation (grey semicircles) and pole elongation pixels (green). **(H)** New cell area is reconstructed by smearing the disk of configured radius along the pixels from F and G yielding white and green area, respectively.

The algorithm requires three parameters to be defined, corresponding to cell width, maximum pole elongation, and the maximal area increase (see Additional file 1 for the precise definition of these parameters and details on how to use them). For any given frame, the algorithm consist of the following steps:

-  Original image (Figure 3A) is processed by applying a 3 x 3 Sobel edge detection filter [24] (Figure 3B).

-  Thresholding is performed using the IsoData algorithm [25] (Figure 3C). Optionally, the user can choose between various other auto threshold algorithms implemented in ImageJ. To assist the user in choosing, BactImAS generates an image stack consisting of the same image frame with the different threshold algorithms applied.
-  Edges are thinned using the Zhang and Suen algorithm [26] (Figure 3D).
-  The resulting image provides information about cell edges used in subsequent steps.
-  Cell selection from the previous frame is copied and thinned to the skeleton [26] (Figure 3E). The skeleton is expended into area by probing whether the previous skeleton pixels and neighboring pixels fit within the configured distance from the cell edge (parameter corresponding to cell width) (Figure 3F). The skeleton is expanded lengthwise at the poles by probing the half disc area oriented away from the skeleton endpoints (Figure 3G). The radius of those discs is determined by the algorithm’s maximum pole elongation parameter. Also, in this step the algorithm is constrained by the configured maximal area increase parameter. Pixel sets obtained in two previous steps form an area used to reconstruct the cell by smearing along it the disk of configured radius (parameter corresponding to cell width) (Figure 3H).

Selections are calculated in a round robin fashion, which is a drawback of this algorithm, as it makes it order dependent. However, this feature comes into play only in situations where there are no detectable borders and it is not clear to which cell to initially assign the joint area.

### Extraction and visualization of quantitative data

The user can extract quantitative data for the annotated cells in a configurable and fully-automated way. Measurements can be taken from images in any file format, as long as they are of the same resolution and alignment as those used in segmentation and tracking (such that ROIs would delineate the same image areas). Additionally, it is possible to define whether to morphologically erode the shape before taking measurement (e.g. for the membrane region to be excluded from the cytoplasmic fluorescence measurements). Furthermore, BactImAS can acquire measurements from a user-defined background area on all channels for each frame. This information is utilized to provide an additional set of background-corrected values for each cell.

Many additional pre-defined variables are provided in the SQLite database to simplify subsequent data analysis, such as interdivision time and growth rate (see Additional file 1: Table S1–S3). The user can export all or a subset of data as a CSV file and then analyze them externally. We provided a built-in SQL editor to query the database and perform basic time-lapse analysis but the user can also use other specialized software such as SQuirreL SQL [27].

BactImAS enables visualization of the cell lineage according to user-defined graphic parameters. In such a lineage tree, the branch length corresponds to the time between divisions and branching points represent division events. If there is more than one cell at the beginning of the experiment, a separate lineage tree is plotted for each cell. Optionally, the designated name of each progeny cell is plotted. The user can select any two variables and visualize them on the lineage tree, one as the branch width and the other as branch color.

## Results and discussion

### BactImAS as a platform for bacterial time-lapse movie analysis

In contrast to other MATLAB-based analysis software [6–10], BactImAS is a freely available, open-source program written in Java. Table 1 lists the major differences between BactImAS and the two most widely-used bacterial time-lapse software Schnitzcells [6] and MicrobeTracker [7]. These two programs fully-automate cell segmentation and tracking by batch processing the movies and then provide limited tools for reviewing and possibly correcting the results. This approach works well if segmentation algorithms rarely fail. However, for many cell types this is not the case and manual intereventions are frequently needed. In contrast, multipurpose image analysis tools such as ImageJ provide full manual control but with limited automation. This manual approach is useful for small datasets, but as the microcolony grows, it quickly becomes error-prone and time consuming. In BactImAS, we combine these two approaches. We automated the straightforward tasks (e.g. cell labeling, extraction of quantitative data) but rely on the user to detect cell divisions and, if the algorithm fails, correct cell segmentation. In doing so, the user retains complete manual control, implemented in an easy-to-use way, and benefits from computer assisted workflow. This semi-automated approach makes BactImAS unique, and well suited for analysis of time-lapse movies in which existing algorithms frequently fail.

**Table 1:** **Comparison of BactImAS with ImageJ** [20]**, MicrobeTracker** [7]**, and Schnitzcells** [6]

	ImageJ	BactImAS	MicrobeTracker	Schnitzcells
Platform	Win/Mac/Linux	Win/Mac/Linux	Win/Mac/Linux	Windows
License	GNU GPL	GNU GPL	GNU GPL + MATLAB license; registration required	Freely available + MATLAB license; registration required
Written in	Java	Java	MATLAB	MATLAB
Data storage	.txt file format	Relational database SQLite3	MATLAB directories	MATLAB directories
ROI storage	Manual, file system	Continous automatic, SQLite3	MATLAB directories	MATLAB directories
Software-user Interaction	Dialog-based GUI	Dialog-based GUI	Dialog-based GUI + command line	Mostly command line
Algorithm optimized for	-	*M. smegmatis*	*C. crescentus*, *E. coli*	*B. subtilis*, *E. coli*
Manual control	Full	Full	Limited	Limited
Cell segmentation	Manual	Semi-automated	Fully-automated	Fully-automated

An important novelty, with respect to all other bacterial time-lapse analysis software, is data storage in the form of a relational database. The advantages of this approach are manifold: easy data backup and exchange, elegant retrieval of results via the standard SQL language, and simple data maintenance/handling in general. We are using the SQLite database as a data repository for all the information extracted from the movies (see database diagram in the Additional file 1). The SQLite is portable, has low memory requirements, and is freely available. However, it is easy to replace SQLite with any other database management system (e.g. MySQL, PostgreSQL) since the Java database connectivity standard is used to connect the program with the database. This replacement would be advisable should the program be configured to use one centralized database. By default, the platform is single-user oriented, with the ability to exchange data with other users by simply transferring a single SQLite file (and associated images).

The BactImAS platform is built in a modular fashion (Figure 1A) to facilitate addition of new functionalities, primarily additional cell segmentation and tracking algorithms. Similar to Schnitzcells [6] and MicrobeTracker [7], it is possible to build an algorithm collection so that users can perform the segmentation and tracking with the best fitting algorithm. To add an algorithm, one has to write a Java class implementing the given programming interface (for detailed description see Additional file 1). BactImAS adopts ImageJ data structures and functionality for various image processing tasks (e.g. edge detection) and adds a number of its own functionalities, which facilitates the development of new algorithms.

### Segmentation and tracking of microfluidics-grown mycobacteria

**Figure 4:**
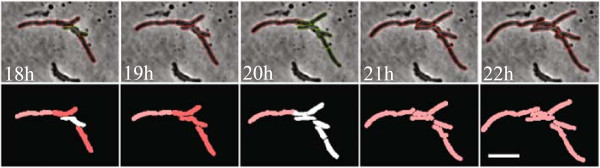
**Segmentation and tracking of mycobacterial cells using BactImAS.** Cell segmentation results are presented as red (automated) and green (manual) outlines with phase-contrast images (top row) and by shaded colors (bottom row) at indicated times. In the bottom row, different stages of descent from a common ancestor (generation) are represented by different nuances of red and manual interventions as white cells. Scale bar, 5 *μ*m.

We validated BactImAS on time-lapse movies of microfluidics-grown *M. smegmatis* cells (Additional file 2). Details on the experimental setup are given in the Additional file 1. The BactImAS algorithm tracked the growth of every individual cell in a microcolony using phase-contrast images with parameters for cell width, maximum pole elongation, and the maximal area increase set to 3, 10, 35, respectively (Figure 4 and Additional file 3). In the presented time-lapse movie, 6.3% (201 out of 3184) of the ROI selections had to be manually corrected. Manual interventions were also required to define division events. However, division events represent only a small percentage (3.7% in the presented movie) of total cell selections in a typical mycobacterial movie. Similar results were obtained with other processed movies, resulting in, at least, a 10-fold decrease of processing time when compared to manual analysis. As these results were obtained using suboptimal images (pixel size was 0.129 *μ*m), we expect a further reduction in the percentage of manual selections when using higher resolution images.

Currently available software solutions were developed for bacterial species where the typical frame-to-frame difference between cells is high, but the programs could rely on the clear cell contours to obtain good segmentation. In our experimental system, the situation is the opposite: cell delineation is difficult, but the tracking is simple because cells grow slowly and minimally change position between two successive frames (Additional file 2). To examine if the developed algorithm is more widely applicable, we processed published time-lapse movies from three different bacterial genera: fast-growing rod-like Bacillus [28], crescent-shaped Caulobacter [7], and filamentous Streptomyces [29]. The most promising results were obtained by processing the Streptomyces time-lapse movie. The main limitation of the algorithm lies in its sensitivity to significant frame-to-frame changes in cell shape and position. Should the skeleton from the previous frame fail to overlap with the contours of the cell body in the following frame, an erroneous selection would likely be generated. This presents a problem when detecting fast-growing organisms (e.g. *E.coli* or *B. subtilis*) and/or when the frequency of imaging is such that significant changes occur between subsequent frames. Hence, selection of an appropriate image acquisition frequency, one that enables good automation of the tracking step, should be considered when designing the time-lapse experiments.

We intend to build a multi-channel version of the algorithm to simultaneously use the information from all available channels. This would potentially allow automated detection of cell division events in mycobacterial strains that express a fluorescently-tagged Wag31protein, a reporter that accumulates at the position of cell division septum and poles [16], clearly marking division events.

### Visualisation and analysis of bacterial gene expression

We illustrate the usage of BactImAS by analyzing real-time expression of *icl*, a gene previously shown to be indispensable for chronic infection of *M. tuberculosis* in mice [30, 31]. A change of carbon source in the growth medium from glucose to acetate affects the quantity and activity of ICL in *M. tuberculosis* [32]. To investigate the role of *icl* induction in this process, we followed the ICL reporter strain of *M. smegmatis* (described in detail in the Additional file 1) in which a green fluorescence protein (GFP) signal serves as a proxy for ICL production within the cell (Figure 2, middle row and Additional file 4). Beside reflecting changes in *icl* expression, the GFP fluorescence is also influenced by fluctuations in the global gene expression machinery (e.g. altered number of ribosomes) within each cell. To account for differences in *icl* expression that stem from these other cellular factors, we monitored, in parallel, the signal of DsRed2, a red fluorescent protein constitutively-expressed from an independent chromosomal locus (Figure 2, bottom row and Additional file 5). Dual-fluorescent ICL reporter cells were cultured in a custom-made microfluidic device [4] and imaged every 10 minutes. For the initial 29 hours cells grew in a glucose-based flow medium and then the glucose was replaced with acetate. We processed three time-lapse movies and extracted quantitative data for every cell of the microcolony. This data is the first such demonstration of single-cell gene expression dynamics in mycobacteria.

**Figure 5:**
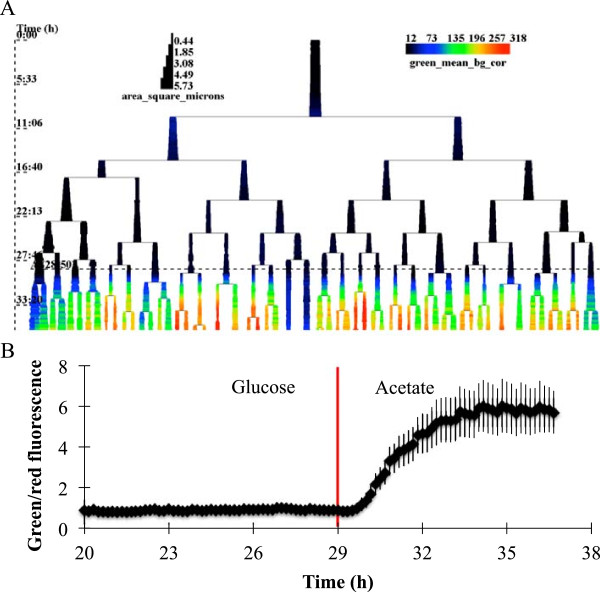
**Visualisation and analysis of gene expression.** **(A)** Lineage tree from the presented time-lapse movie. Color scale represents background-corrected mean green fluorescence in arbitrary units (AU). Cell area (*μ*m^**2**^) is displayed as proportional to the branch width. Time is indicated on the vertical axis. Switch to the acetate-based carbon source of the growth medium (dashed horizontal line) induces strong expression of GFP, representing induction of the *icl* gene. **(B)** The average green-to-red fluorescence ratio calculated from all cells from three time-lapse movies. Throughout the course of the experiment there were 299 cells in total. Error bars represent standard deviation. Red vertical line indicates the time of switch of the carbon source in growth media from glucose to acetate.

Although time-lapse data is commonly represented as a lineage tree, researchers predominantly use unavailable in-house software. BactImAS includes a novel and advanced visualization tool that can display lineage tree(s) superimposed with any two variables from the database. A representative tree, with cell size displayed as proportional to the branch width and mean green fluorescence mapped to a color scale, demonstrates the advantages of such visualization for representing complex information (Figure 5A). Upon switching to an acetate-based growth medium, there is a clear increase in *icl* expression, with concomitant decrease in typical cell size. Phenotypic heterogeneity in *icl* induction and cell growth, observed even between sibling cells, is clearly evident from such visualization.

The built-in SQL editor allows data analysis to be performed directly with BactImAS. With a single query to the database (see Additional file 1), we analyzed the dynamics of *icl* expression in all processed movies. We calculated the green/red fluorescence ratio for every cell (7306 ROIs) and then plotted the average ratio as a function of time (Figure 5B). The *icl* expression was found to be stable in the 9 hour interval prior to the glucose-to-acetate switch. Upon the switch, *icl* expression continuously increased over the next 5 hours before reaching a 6-fold higher plateau. This is in very good agreement with the 5.9-fold difference in the *icl* expression level determined after 24 hours by quantitative real-time PCR in *M. tuberculosis* [32].

## Conclusion

Here we present BactImAS – a Java-based, open-source platform for semi-automated analysis of bacterial time-lapse movies. Image processing tasks are carried out through a user-friendly GUI and incorporated ImageJ program. All resulting information is stored and retrieved from an SQLite database. The platform includes a newly-developed segmentation and tracking algorithm based on the assumption that the frame-to-frame difference in a cell’s shape and size is usually very small. The algorithm allowed us to process mycobacterial time-lapse movies for the first time. While the user is still responsible for identifying cell division events, we were able to reduce the number of manual selections by 90%. Finally, we implemented a novel graphic tool and an SQL editor and illustrate their usefulness by analyzing *icl* gene expression in *M. smegmatis*, the first such gene expression study in the Mycobacterium genus.

The BactImAS platform is currently the only available tool for analysis of mycobacterial time-lapse movies and we believe it will be useful for other similar organisms. By adding segmentation algorithms optimized for other bacterial species, it has the potential to become a much-needed platform for universal bacterial time-lapse movie analysis.

## Availability and requirements

**Project name**: BactImAS **Project home page**: http://homer.zpr.fer.hr/BactImAS/** Operating system(s)**: Linux, MacOS X, Windows **Programming language**: Java **Other requirements**: Java 1.6 or higher and SQLite3 **License**: GNU GPL v3. Please cite this paper in any publications that use this software. **Any restrictions to use by non-academics**: None.

## Electronic supplementary material

Additional file 1: **Details about software implementation and experimental setup.** 1) Algorithm used to correct translational shift between frames; 2) Algorithm for segmentation of mycobacteria-like cells; 3) Algorithm parameters; 4) List of variables measured and calculated by BactImAS; 5) Implementation of a new algorithm; 6) Relational model diagram of the BactImAS database; 7) Details about experimental setup; 8) SQL query used to obtain Figure 5B. (PDF 715 KB)

Additional file 2: **Time-lapse movie of** ***M. smegmatis*** **acquired on phase-contrast channel.** Details about the experimental setup are presented in Additional file 1. (MOV 7 MB)

Additional file 3: **Cell segmentation results superimposed on phase-contrast movie of** ***M. smegmatis*** **.** Manual interventions (ROI corrections and annotation of newly divided cells) are indicated with a green outline and automatic ones with red outline. (MOV 14 MB)

Additional file 4: **Time-lapse movie of** ***M. smegmatis*** **acquired on green fluorescence channel.** GFP was used as a proxy for *icl* expression during switch of the carbon source in growth media from glucose to acetate. (MOV 4 MB)

Additional file 5: **Time-lapse movie of** ***M. smegmatis*** **acquired on red fluorescence channel.** This channel was used to monitor expression of DsRed2, a red fluorescent protein constitutively-expressed from an independent chromosomal locus, during switch of the carbon source in growth media from glucose to acetate. (MOV 8 MB)
