# Supplementary material for: BactImAS: a platform for processing and analysis of bacterial time-lapse microscopy movies
Source: BMC Bioinformatics. 2014 Jul 25;15(1):251. doi: 10.1186/1471-2105-15-251 (PMC4122790; doi:10.1186/1471-2105-15-251)
Supplement: Supplementary file 1 — Additional file 1: Details about software implementation and experimental setup. 1) Algorithm used to correct translational shift between frames; 2) Algorithm for segmentation of mycobacteria-like cells; 3) Algorithm parameters; 4) List of variables measured and calculated by BactImAS; 5) Implementation of a new algorithm; 6) Relational model diagram of the BactImAS database; 7) Details about experimental setup; 8) SQL query used to obtain Figure 5B. (PDF 715 KB) [file 12859_2014_6521_MOESM1_ESM.pdf]

Additional file 1 for **BactImAS: a platform for processing and analysis of bacterial time-lapse microscopy movies**

Igor Mekterović, Darko Mekterović and Željka Maglica

1 Algorithm used to correct translational shift between frames

BactImAS has a simple and robust built-in algorithm to register images. This algorithm is defined as follows: let $I_n(i,j)$ be the image at frame (time) n and $S_n = F(I_n)$ be the binary image created by applying edge detection (3 x 3 Sobel filter) [1] and IsoData threshold algorithm [2] to the image I_n. For the frame n, we find the translation vector (x, y) by maximizing the following expression:

$$\sum_{i,j}(S_{n-1}(i,j) \cdot S_n(i+x, j+y)) \quad (1)$$

where $-r \leq x \leq r$ and $-r \leq y \leq r$, that is, by sliding the transformed image r pixels in all directions and finding the maximum sum of the element-wise products of binary images. The number of steps (in pixels) scanned within each image is user-defined.

2 Algorithm for segmentation of mycobacteria-like cells

We based our algorithm on the following assumptions:

(i) Cells exhibit only slight changes from one frame to the other (retain similar area and position) thus making the tracking straightforward

(ii) Cells are sometimes in close juxtaposition to neighboring cells but most juxtaposed cells have at least some segments of visible edge

(iii) Cells are worm-shaped

(iv) Cells grow mostly lengthwise (retain a fixed or similar diameter)

Following on assumption (i), the algorithm copies the shapes of cells from the previous frame, applies them to the current frame, and tries to adjust them so they would fit well with detectable edges (ii). This requires for the initial state $R_1 = \{r_{1,i}\}$ to be defined by the user, where $r_{1,i}$ represents ROI of the cell i on the frame one. For a given frame, cells are detected using the previous state, translation vector t_n, and the current image: $R_n = f(R_{n-1}, t_n, I_n)$. To compute the r_i on the frame n we take the following steps. Initialize $r_{n,i} = translate(r_{n-1,i}, t_n)$ by translating the previous ROIs using the translation vector t_n. For frame n with regards to each $r_{n,i}$ we define the following penalty images (masks):

$$P_{n,i}(p) = \begin{cases} -\infty, & \text{where pixel } p \in r_{n,j} \text{ and } j \neq i \\ 0, & \text{otherwise} \end{cases} \tag{2}$$

that will be used to prevent adjacent cells from claiming each other's area. Image $I' = f'(I)$ is then calculated (Fig. S1A) by applying edge detection (3x3 Sobel edge detection [1]), thresholding (configurable automatic threshold algorithm, IsoData [2] by default), and skeletonization (the Zhang and Suen thinning algorithm [3]). With $r_{n,i}$, $P_{n,i}$ and I' we proceed as follows with our copy-and-adjust algorithm: Skeletonize r_i and apply it to I'. We now have a skeleton of the previous selection mapped to the new image I'.

Following on the assumption (iv) we try to:

(a) slightly adjust the outline of selection along its axis and

(b) expand the selection lengthwise

This is performed using a filtering or convolution-like operation with mask (kernel) B. We define B as square matrix of $(2*r+1)$ dimensions, where r is a configurable parameter corresponding to the bacteria width:

$$B = [b_{i,j}] \text{ where } b_{i,j} = \begin{cases} 1, & \text{where } (r-i)^2 + (r-j)^2 = r^2 \\ -1, & \text{where } (r-i)^2 + (r-j)^2 < r^2 \\ 0, & \text{otherwise} \end{cases} \tag{3}$$

In other words, B is a circle shaped mask having 1 on the rim and -1 inside the circle (Fig. S1B). To achieve (a) the algorithm calculates the new skeleton by using sets A and A_c, defined as:

$$A = \{p\colon p \in N_{24}(skeleton(r_i)) \wedge \ apply(p, B, I', P_{i,n}) > 0\} \tag{4}$$

where $N_{24}(skeleton(r_i))$ is a set of 24-neighbors (5 x 5 kernel) of r_i's skeleton and *apply* is:

$$apply(q, B, I, P) = \sum_{i,j=0}^{2r+1} b_{i,j} \cdot i_{i+q_x-r,j+q_y-r} \cdot p_{i+q_x-r,j+q_y-r} \tag{5}$$

that is, we perform a filtering operation where a pixel is kept (added to the set A) if the sum of multiplied corresponding mask, image, and penalty values yield a positive result (Fig. S1C). This allows B to "touch" the edge of the shape from within, but not to cross it (as negative weights in the mask discourage that), while avoiding the area already taken by other cells (penalty mask). This filtering operation can result in a set of disconnected points (especially in images with missing edges), so we define the set of connected points A_c as:

$$A_c = \{p\colon p \in convexHull(A)\} \tag{6}$$

We have used [4] to calculate the convex hull of A. To achieve (b) we search for possible skeleton lengthening points by finding the set E, defined as:

$$E = \{e\colon e \in awayPoints(r_{n,i}) \wedge \ apply(e, B, I', P_{n,i}) > 0\} \tag{7}$$

where (Fig. S1D):

$$awayPoints(r_{n,i}) = \{a\colon \delta(a, e) < M \wedge \ \delta(a, e) < \delta(a, N_s(e, r_{n,i}))\} \tag{8}$$

where δ denotes Euclidian distance, M is a constant (e.g. $2.5 \cdot r$), e is one of the two endpoints of $skeleton(r_{n,i})$ and $N_s(e, r_{n,i})$ is the neighbor of endpoint e that is also a member of the $skeleton(r_{n,i})$ (Fig. S1D).

Finally, the new selection is built using the two sets, A_c and E (Fig. S1E):

$$r_{n,i} = \{p\colon p \in mask(B, p) \wedge \ p \in A_c \cup E\} \tag{9}$$

where

$$mask(B, p) = \{q\colon q \in B_p \wedge \ B_p(q) \neq 0\} \tag{10}$$

where B_p is B whose center is translated to the point p.

That is, we create the new selection by making a union of disks of radius r having a center in $A_c \cup E$. First, set A_c is used to reconstruct the cell, and then the area based on pixels from A_c is added, minding not to exceed the configured 'maximal area increase' parameter. To that end, we sort the pixels for A_c with regards to their distance from the skeleton endpoints and add their contributions, one by one, checking the new cell size at each step.

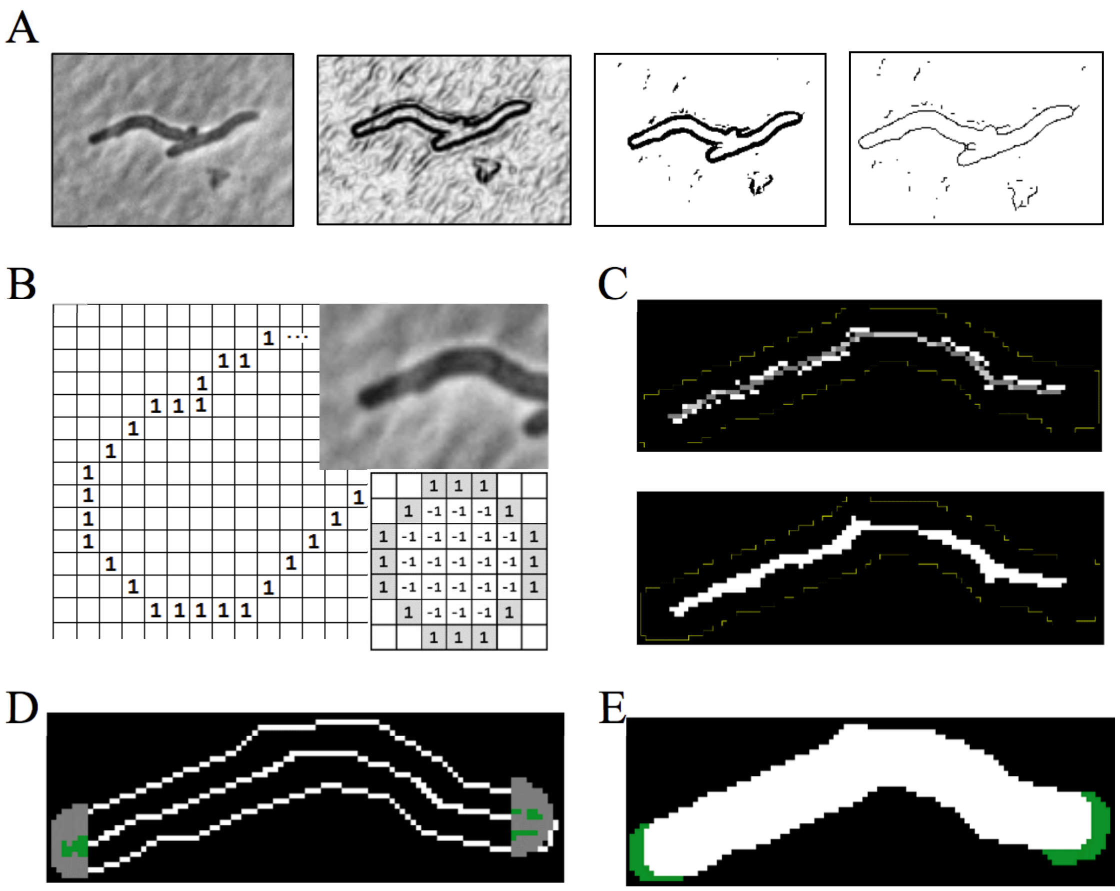

Figure S1: Algorithm description (A) Computing I' from I: starting from the original image, we apply the following transformations: edge detection, thresholding, and skeletonization (to acquire I'). (B) Constructing the new selection. Partial ideal I' for the image I shown in upper right corner. Non-edge pixel values have a value of zero, omitted here for clarity. Mask (kernel) having r=3 shown on the bottom right side. When applied to the ideal I', the filter operation will keep the pixels where the mask "touches" the borders of the cell and never crosses them due to the inner negative (-1) weights. (C) A and Ac sets, as defined in equations (4) and (6). (D) gray half-disk showing awayPoints set, as defined in (8). Set E (7) shown in green. (E) New cell selection, with contributions from A_c and E shown in white and green, respectively

3 Algorithm parameters

- Cell width (called ”Ball radius” in the GUI dialog) is a value (in pixels) that defines the radius of the disk used to draw the cell. You can measure width W by using ImageJ and then assign the ball radius to a value just below the W/2. The default value is 4, which is appropriate for cells of 9 to11 pixels in width.

- Maximum pole elongation (called ”Range” in the GUI dialog) is a value (in pixels) related to the possible elongation of the cells at their poles. This number should be determined by comparing cell elongation between two subsequent frames as this parameter increases with higher image resolution and faster growth rates. The default is set to 20 pixels.

- Maximal area increase (called ”Max ball delta perc” in the GUI dialog) is given in percentages and defines the maximal additional area one disk can contribute when expanding the selection. The default is set to 35 but it should be decreased if cell selections are erroneously growing too fast, ”bubbling up” on the sides and poles.

References

[1] Sobel. http://homepages.inf.ed.ac.uk/rbf/HIPR2/sobel.htm

[2] Ridler, T.W., Calvard, S.: Picture thresholding using an iterative selection method. IEEE Trans. System, Man and Cybernetics **8**, 630–632 (1978)

[3] Zhang, Y., Suen, C.Y.: A fast parallel algorithm for thinning digital patterns. Commun. ACM **27**, 236–239 (1984)

[4] Duckham, M., Kulik, L., Worboys, M., Galton, A.: Efficient generation of simple polygons for characterizing the shape of a set of points in the plane. Pattern Recogn. **41**, 3224–3236 (2008)

4. List of variables measured and calculated by BactImAS

The user can define which parameters will be saved to the database. They can be divided into three groups: defined by ImageJ (Table S1), defined or set by default by BactImAS (Table S2), and pre-defined variables calculated by the SQLite database (Table S3).

Table S1. Most frequently used variables measured with ImageJ.

Name	Description	Visualization
area	Measured area	
red_mean	Mean red fluorescence of segmented cell	✔
green_mean	Mean green fluorescence of segmented cell	✔
blue_mean	Mean blue fluorescence of segmented cell	✔
red_bg	Mean red background fluorescence	✔
green_bg	Mean green background fluorescence	✔
blue_bg	Mean blue background fluorescence	✔

Table S2. Parameters defined by BactImAS

Name	Description	Visualization
exp_name	User defined experiment name	
id_experiment	Program defined experiment identification	
frame_no	Frame number	
seconds_per _frame	User defined time between frames	
bact_name	User defined name of segmented cell	✔
id_bacteria	Program defined identification of segmented cell	

Table S3. Pre-defined variables calculated by the SQLite database

Name	Description	Vis.
time_s	=frame_no * (seconds per frame)	✔
generation	Stage of descent from a common ancestor	
parent	Mother cell	
sibling	Sibling cell derived from the same mother cell	
progeny_a	Daughter cell a	
progeny_b	Daughter cell b	
birth_time	Cell's birth time	✔
division_time	Cell's division time	✔
interdivision_time	= division_time - birth_time	✔
growth_rate	=(area at division_time/area at birth_time)/interdivision_time	✔
growth_velocity	=(area at division_time - area at birth_time)/interdivision_time	✔
area_square_microns	= area* (pixel_width*pixel_height)/scale*scale	✔
area_square_microns_growth	=(area_square_microns(n)-area_square_microns(n-1))/time_s	✔
red_mean_bg_cor	=red_mean-red_bg	✔
green_mean_bg_cor	=green_mean-green_bg	✔
blue_mean_bg_cor	=blue_mean-blue_bg	✔
red_ctcf	=red_mean_bg_cor*area_square_microns	✔
green_ctcf	=green_mean_bg_cor*area_square_microns	✔
blue_ctcf	=blue_mean_bg_cor*area_square_microns	✔
red_mean_bg_cor_change	=(red_mean_bg_cor (n)-red_mean_bg_cor (n-1))	✔
green_mean_bg_cor_change	=(green_mean_bg_cor(n)-green_mean_bg_cor(n-1))	✔
blue_mean_bg_cor_change	=(blue_mean_bg_cor (n)-blue_mean_bg_cor (n-1))	✔
red_ctcf_change	=(red_ctcf(n)-red_ctcf(n-1))	✔
green_ctcf_change	=(green_ctcf(n)-green_ctcf(n-1)	✔
blue_ctcf_change	=(blue_ctcf(n)-blue_ctcg(n-1))	✔

5. Implementation of a new algorithm

To add a new algorithm, the developer has to do two things:

(a) Write a class that implements the `ITrackingAlgorithm` interface (javadoc at: http://homer.zpr.fer.hr/bactimas/doku.php?id=javadoc:javadoc).

These are the summaries of the requested methods:

`void beforeBatch(int firstFrameNo)`
Invoked before processing batch.
`void beforeStep(int frameNo)`
Invoked before every step().
`java.lang.String getAbbrev()`
Algorithm name abbreviation, used for display in the frame tree.
`java.lang.String getClassName()`
Class name, used for instantiation.
`java.lang.String getName()`
A friendly name, to be shown to the user.
`void step(int frameNo, VPoint translation)`
This is the main function to implement - the one that performs the tracking segmentation.

From within these methods, primarily `step()` method, a developer has at his disposal the entire ImageJ library and several additional BactImAS methods, such as (from the `CurrentExperiment` class):

`ImagePlus getImagePlus(ImageStripType channel, int frameNo, String altFormat)`
Returns the ImageJ's ImagePlus object for the given frameNo, channel, and (not required) alternative image format (tif, jpg, ...) with png being the default
`LinkedList<Bacteria> getBacteriasForFrame(int frameNo)`
Returns the list of bacterial cells on the given frame.
`boolean saveROI(Roi roi, int frameNo, Bacteria b, int roiType)`
Persists the (calculated) ROI for the bacteria b into the database.
etc.

Also, it is probably helpful to take a look at the implementation of the proposed "Copy And Adjust" algorithm in the class:

```
hr.fer.zpr.algorithms.CopyAndAdjustAlgorithm implements
ITrackingAlgorithm
```

(b) Append the full class name in the conf/all.algorithms file text file so that BactImAS can instantiate it at run time.

6. Relational model diagram of the BactImAS database. Views are shown in gray:

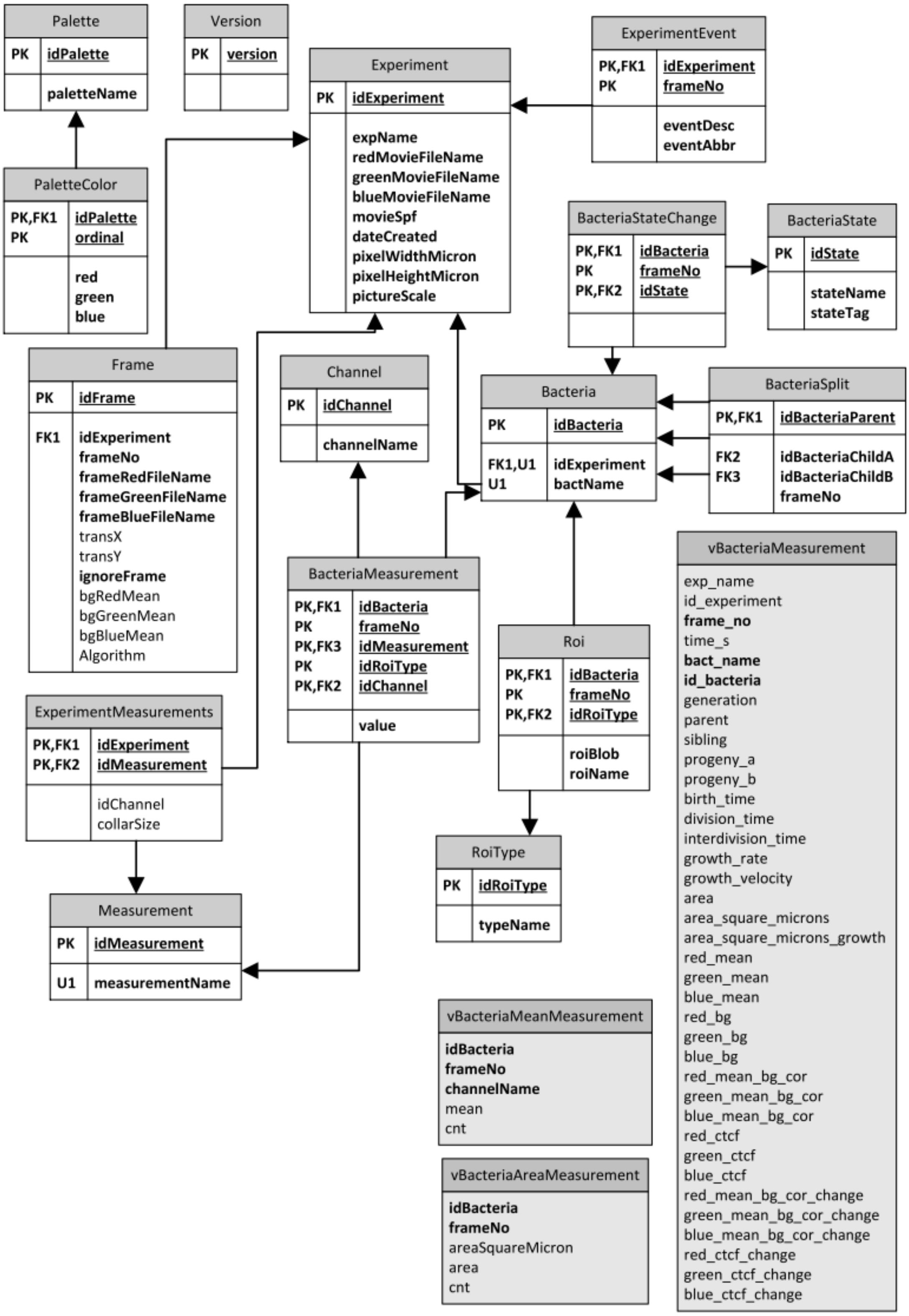

7. Dataset used

Gene expression dynamics of the *icl* gene was monitored by time-lapse fluorescence microscopy in the model organism *M. smegmatis*. A gene for GFP (Green fluorescent protein) was introduced 5 base pairs downstream of the chromosomal *icl* stop codon, without disrupting neighboring genes [1, 2]. In this way, a single RNA including both *icl* and *GFP* is produced upon gene activation. This RNA is then used by the ribosome to produce two separate proteins: ICL and GFP. Thus, green fluorescence is a proportional proxy for production of the ICL protein. However, intensity of fluorescence can also depend on the environmental conditions. As a control that change in green fluorescence derives predominantly from the *icl* induction, red fluorescent protein (DsRed2) was simultaneously expressed independently from a constitutive UV15 promoter at a different chromosomal position (*attB*) (pND239 kindly provided by N. Dhar). To monitor these two fluorescent proteins, images were acquired in two fluorescent channels in addition to phase-contrast images. The change of carbon source in the growth medium from glucose to acetate affects the quantity and activity of ICL in *M. tuberculosis* [3]. To investigate the role of *icl* induction in this process, the *M. smegmatis* reporter strain was grown in M9 minimal medium supplemented with either glucose or acetate. Specifically, bacteria were grown at 37 °C in medium consisting of: 1X M9 salts (Sigma), 2mM $MgSO_4$, 0.1 mM $CaCl_2$, and either 0.5% glucose (w/v) or 0.1% acetate (w/v). Use of minimal medium results in slower growth and division rates (doubling time 5-6h), compared to those observed in rich growth medium (7H9, doubling time 3.4h)[4].

For time-lapse experiments, bacteria were cultured in a custom-made PDMS-based microfluidic device with continuous flow of growth medium [4]. Bacteria were imaged using a DeltaVision personalDV inverted fluorescence microscope (Applied Precision) equipped with a 100x oil immersion objective (Olympus Plan Semi Apochromat, 1.3 N.A.), and an environmental chamber maintained at 37°C. Images were acquired at 10 minute intervals in 256x256 size with 2x2 binning (pixel size = 0.129 µm) on phase-contrast and fluorescence channels (for GFP, excitation filter 490/20, emission filter 528/38; for DsRed2, excitation filter 575/25, emission filter 632/60) using a CoolSnap HQ2 camera (Fig. 1 and Supplementary Movies 1-3). Small imaging area and binning was necessary in order to increase the number of focused images in this long-term experiment (software-based autofocus is used).

REFERENCES:

1. Parish T, Stoker NG: **Use of a flexible cassette method to generate a double unmarked Mycobacterium tuberculosis tlyA plcABC mutant by gene replacement**. *Microbiology* 2000, **146 (Pt 8)**:1969-1975.
2. Pavelka MS, Jr., Jacobs WR, Jr.: **Comparison of the construction of unmarked deletion mutations in Mycobacterium smegmatis, Mycobacterium bovis bacillus Calmette-Guerin, and Mycobacterium tuberculosis H37Rv by allelic exchange**. *J Bacteriol* 1999, **181**(16):4780-4789.
3. Micklinghoff JC, Breitinger KJ, Schmidt M, Geffers R, Eikmanns BJ, Bange FC: **Role of the transcriptional regulator RamB (Rv0465c) in the control of the glyoxylate cycle in Mycobacterium tuberculosis**. *J Bacteriol* 2009, **191**(23):7260-7269.
4. Wakamoto Y, Dhar N, Chait R, Schneider K, Signorino-Gelo F, Leibler S, McKinney JD: **Dynamic persistence of antibiotic-stressed mycobacteria**. *Science* 2013, **339**(6115):91-95.

8. SQL query used to obtain figure 5B

Query aggregates the values from three experiments (ids 1076, 1077 and 1078).

```
SELECT fn, time_s,
  (SELECT AVG(green_mean_bg_cor/red_mean_bg_cor)
     FROM vBacteriaMeasurement
    WHERE frame_no =  fn
      AND  id_experiment IN (1076, 1077, 1078)) AS GFP_dsRED _ratio,
  (SELECT COUNT(*)
     FROM vBacteriaMeasurement WHERE frame_no =  fn
      AND  id_experiment IN (1076, 1077, 1078) ) as cnt
   FROM
   (SELECT DISTINCT frame_no as fn, time_s
      FROM vBacteriaMeasurement
     WHERE id_experiment IN (1076, 1077, 1078)
     ) AS frames

ORDER BY fn
```
